# Supplementary material for: Genome-wide mapping of fluoroquinolone-stabilized DNA gyrase cleavage sites displays drug specific effects that correlate with bacterial persistence
Source: Nucleic Acids Res. 2023 Jan 12;51(3):1208–28. doi: 10.1093/nar/gkac1223 (PMC9943676; doi:10.1093/nar/gkac1223)
Supplement: gkac1223_Supplemental_Files [file gkac1223_supplemental_files.zip › Supplementary_Tables_11.29.22.pdf]

Supplementary Table S1. Bacterial strains and plasmids

**Strain**	**Relevant genotype**	**Source or Reference**
MG1655	F-, λ-, *ilvG-*, *rfb-50*, *rph-1*	ATCC 700926 (1)
Mu_origin 1	MG1655 *pstS-glmS*::*cat-Mu*	This work; Integration of 1241bp of *cat*-Mu in the intergenic region between *pstS* and *glmS* using the method of Datsenko and Wanner (2)
Mu_origin 1_gyrA-FLAG	Mu_origin 1 *gyrA-FLAG-kan*	This work; Integration of FLAG-*kan* at C-terminus of *gyrA* using Datsenko and Wanner method (2)
Mu_origin 1_gyrA-FLAGless	Mu_ origin 1 *gyrA-kan*	This work; Integration of *kan* at C-terminus of *gyrA* using Datsenko and Wanner method (2)
Mu_origin 2	MG1655 *aptI-rsmG*::*Mu*	This work; Integration of Mu-*kan* in the intergenic region between *aptI* and *rsmG* using Datsenko and Wanner method and cured of *kan* using pCP20 (2)
Mu_terminus 1	MG1655 *yddY-yddW*::*Mu*	This work; Integration of Mu-*kan* in the intergenic region between *yddY* and *yddW* using Datsenko and Wanner method and cured of *kan* using pCP20(2)
Mu_terminus 2	MG1655 *ydiY-pfkB*::*kan-Mu*	This work; Integration of Mu-*kan* in the intergenic region between *ydiY* and *pfkB* using Datsenko and Wanner method (2)
MuScr_origin 1	MG1655 *pstS-glmS*::*cat-MuScr*	This work; Integration of 1241bp of *cat*-MuScr (scrambled sequence) in the intergenic region between *pstS* and *glmS* using Datsenko and Wanner method (2)
MuScr_origin 1_gyrA-FLAG	Mu Scrambled_origin1 *gyrA-FLAG-kan*	This work; P1 phage transduction of FLAG-*kan* at C-terminus of *gyrA* from Mu_origin 1_gyrA-FLAG

MuScr_origin 2	MG1655 *aptI-rsmG*::*MuScr*	This work; Integration of MuScr-*kan* in the intergenic region between *aptL* and *rsmG* using Datsenko and Wanner method and cured of *kan* using pCP20 (2)
MuScr_terminus 1	MG1655 *yddY-yddW*::*MuScr*	This work; Integration of MuScr-*kan* in the intergenic region between *yddY* and *yddW* using Datsenko and Wanner method and cured of *kan* using pCP20 (2)
MuScr_terminus 2	MG1655 *ydiY-pfkB*::*kan-MuScr*	This work; Integration of MuScr-*kan* in the intergenic region between *ydiY* and *pfkB* using Datsenko and Wanner method (2)
MG1655 Δ*recD*	MG1655 Δ*recD*	This work; P1 phage transduction of mutation from Keio collection followed by curing of the kanamycin resistance cassette (3)
PIR1	F- Δ*lac169 rpoS(Am) robA1 creC510 hsdR514 endA recA1 uidA(*Δ*MluI)::pir-116*	Invitrogen; The *pir* gene in PIR1 encodes the replication protein *π*, which is required to replicate and maintain plasmids containing the R6Kγ origin. The strain is used to maintain pKD3_Mu, pKD4_Mu, pKD3_MuScr, pKD4_MuScr used in this study
MG1655 4Mu_gyrA-FLAG	Mu_ origin 1 *yddY-yddW*::*Mu ydiY-pfkB*:: *Mu aptI-rsmG*::*Mu gyrA-kan*	This work; Integration of Mu-*kan* using sequential P1 transduction of Mu_terminus 2, Mu_origin 2, and Mu_origin 1_gyrA-FLAG to Mu_terminus 1 strain with step-wise curing of the kanamycin resistance cassette(3)
**Plasmid**	**Description**	**Source**
pKD3	Plasmid conferring chloramphenicol and ampicillin resistance	(2)

pKD4	Plasmid conferring kanamycin and ampicillin resistance	(2)
pKD3_Mu	Mu strong gyrase cleavage sequence cloned into pKD3	This work
pKD3_MuScr	Mu Scrambled sequence cloned into pKD3	This work
pKD4_Mu	Mu strong gyrase cleavage sequence cloned into pKD4	This work
pKD4_MuScr	Mu Scrambled sequence cloned into pKD4	This work
pKD4_gyrA-FLAG	C-terminus of FLAG-tagged *gyrA* cloned into pKD4	This work
pCP20	pCP20, repA101(ts), Amp^R and CAM^R	(2)

Supplementary Table S2. DNA oligonucleotides

Primers/gBlocks for plasmid construction		
**Primer name**	**Sequence**	**Description**
Mu _Gibson_pKD3	GATCTTCCGTCACAGGTAGGATGTGCTGC AAGGCGATTAAGTTGGGTAACGCCAGGG TTTTCCCAGTCACGACGTTGTAAAACGAC GGCCAGTGCCAAGCTTGCATGCCTGCAG GTCACTGGAGAAAGAAAGTGAAAGGAA GATAAAACGGGATTCATACACCGTTAAA TACCGGTTTAAAAATCCCGTGGCGCGTTT TAAAAAATCTGTGCGGGTGATTTTATGCC TGATTCTGTTTATTGCCTCAGAGCGGCGC TGACGCGTTTTCTGATGGCATCAAAAATT TCCTGTTCCCCGGTCTTATCCAGCCCCAT ATAAGGACGCGCAGGAACGCCTGCCGGG CGGGGTGCCATATCGGGTGTACCGCCCTC CCATGTCAGCCGTTAAGT	389bp gBlock gene fragments containing the strong gyrase cleavage site Mu (4) for the construction of pKD3_Mu
MuScr_pKD3_1	GATCTTCCGTCACAGGTAGGATGTGCTGC AAGGCGATTAAGTTGGGTAACGCCAGGG TTTTCCCAGTCACGACGTTGTAAAACGAC GGCCAGTGCCAAGCTTGCATGCCTGCAG GTCACTGGAGAAAGAAAGTGAAAGGAA GATAAAACGGGATTCATACACCGTTAAA TACCGGTTTAAAAATCCCGTGGCGCGTA GTCTTGATTTTAGAGTCTGAGCCGAGTGC GGTTTCATTTCCCGGGATAGTGGTTCAGA AACCTCTTTTTTCTGATGGCATCAAAAAT TTCCTGTTCCCCGGTCTTATCCAGCCCCA TATAAGGACGCGCAGGAACGCCTGCCGG	389bp gBlock gene fragments containing the Mu scrambled sequence for the construction of pKD3_MuScr

	GCGGGGTGCCATATCGGGTGTACCGCCC TCCCATGTCAGCCGTTAAGT	
pKD3_FWD	TCCCATGTCAGCCGTTAAGTG	Used in conjunction with pKD3_REV to amplify the backbone of pKD3 or pKD4_REV to amplify the backbone of pKD4
pKD3_REV	CCTACCTGTGACGGAAGATCAC	Used in conjunction with pKD3_FWD to amplify the backbone of pKD3
Cat_R	GCAACTGACTGAAATGCCTC	Used to confirm insertion of Mu or MuScr in pKD3 by Sanger sequencing
Cam_pKD3_ext_rev	AAGCAGAAGGCCATCCTGAC	Used to confirm insertion of Mu or MuScr in pKD3 by Sanger sequencing
cmR_int_fwd_1	TCGTCTCAGCCAATCCCTG	Used to confirm insertion of Mu or MuScr in pKD3 by Sanger sequencing
Mu_ext_rev	CGAACTAAACCCTCATGGC	Used to confirm insertion of Mu or MuScr in pKD3 or pKD4 by Sanger sequencing
Mu_pKD4	GAGGATATTCATATGGACCAATGTGCTG CAAGGCGATTAAGTTGGGTAACGCCAGG GTTTTCCCAGTCACGACGTTGTAAAACGA CGGCCAGTGCCAAGCTTGCATGCCTGCA GGTCACTGGAGAAAGAAAGTGAAAGGA AGATAAAACGGGATTCATACACCGTTAA ATACCGGTTTAAAAATCCCGTGGCGCGTT TTAAAAAATCTGTGCGGGTGATTTTATGC CTGATTCTGTTTATTGCCTCAGAGCGGCG CTGACGCGTTTTCTGATGGCATCAAAAAT TTCCTGTTCCCCGGTCTTATCCAGCCCCA TATAAGGACGCGCAGGAACGCCTGCCGG GCGGGGTGCCATATCGGGTGTACCGCCC TCCCATGTCAGCCGTTAAGT	389bp gBlock gene fragments containing the strong gyrase cleavage site Mu (4) for the construction of pKD4_Mu

MuScr_pKD4	GAGGATATTCATATGGACCAATGTGCTG CAAGGCGATTAAGTTGGGTAACGCCAGG GTTTTCCCAGTCACGACGTTGTAAAACGA CGGCCAGTGCCAAGCTTGCATGCCTGCA GGTCACTGGAGAAAGAAAGTGAAAGGA AGATAAAACGGGATTCATACACCGTTAA ATACCGGTTTAAAAATCCCGTGGCGCGT AGTCTTGATTTTAGAGTCTGAGCCGAGTG CGGTTTCATTTCCCGGGATAGTGGTTCAG AAACCTCTTTTTTCTGATGGCATCAAAAA TTTCCTGTTCCCCGGTCTTATCCAGCCCC ATATAAGGACGCGCAGGAACGCCTGCCG GGCGGGGTGCCATATCGGGTGTACCGCC CTCCCATGTCAGCCGTTAAGT	389bp gBlock gene fragments containing the Mu scrambled sequence for the construction of pKD4_MuScr
pKD4_SEQ_FWD	GCCATCACGAGATTTCGATT	Used to confirm insertion of Mu or MuScr by Sanger sequencing
gyrA_CFLAG	GAGGATATTCATATGGACCATTACTTGTC ATCGTCGTCCTTGTAGTCGGATCCTTCTT CTTCTGGCTCGTCGTCAACGTCCACTTCC GGAGCGATTTCATCGTCCCCTTCCGCTCC CATGTCAGCCGTTAAGTG	Gene fragments containing the FLAG tag to the C-terminus of GyrA; Used for insertion into pKD4 next to the *kan* cassette
pKD4_REV	TGGTCCATATGAATATCCTCCTTAG	Used in conjunction with pKD3_FWD to amplify pKD4 backbone
**Primers for chromosomal perturbations**		
**Primer name**	**Sequence**	**Description**
cat_Mu_pstS_pkD3_FWD	TAAGCGTTGATATTCAGTCAATTACAAAC ATTAATAACGAGGAATAGGAACTTCATT TAAATGGCGCG	Used in conjunction with cat_Mu_glmS_pKD3_REV to amplify Mu or MuScr from pKD3_Mu or pKD3_MuScr for

		construction of Mu_origin 1 or MuScr_origin 1
cat_Mu_glmS_pKD3_REV	CTTTTTCTCTGTCACAGAATGAAAATTTTTCTGTCATCTCTGGGCGGTACACCCGATATG	Used in conjunction with cat_Mu_pstS_pkD3_FWD to amplify Mu or MuScr from pKD3_Mu or pKD3_MuScr for construction of Mu_origin 1 or MuScr_origin 1
gyrA_CFLAG_FWD	CAATTCAAACAAGGGAGATAGCTCCCTTTTGGCATGAAGAAGTAAAAGTGTAGGCTGGAGCTGCTTCG	Used in conjunction with gyrA_CFLAG_REV to amplify *gyrA-FLAG* from pKD4_gyrA-FLAG for construction of Mu_origin 1-gyrA-FLAG
gyrA_CFLAG_REV	GCGGAAGGGGACGATGAAATC	Used in conjunction with gyrA_CFLAG_FWD to amplify *gyrA-CFLAG* from pKD4_gyrA-FLAG for construction of Mu_origin 1-gyrA-FLAG
pKD4_rev_gyrA	GATGAAATCGCTCCGGAAGTGGACGTTGACGACGAGCCAGAAGAAGAATAATGGTCCATATGAATATCCTCCTTAG	Used in conjunction with gyrA_CFLAG_FWD to amplify *gyrA-kan* from pKD4 for construction of Mu_origin 1-gyrA-FLAGless
rsmG_Mu_FWD	GTTTTAATAAATGACATTTACACAACAAAAACCACCCATTGAGTGTAGGCTGGAGCTGCTTCG	Used in conjunction with rsmG_Mu_REV to amplify Mu or MuScr from pKD4_Mu or pKD4_MuScr for construction of Mu_origin 2 or MuScr_origin 2
rsmG_Mu_REV	CTAAGAACCATCATTGGCTGTTAAAACATTATTAAAAATGGGGCGGTACACCCGATATG	Used in conjunction with rsmG_Mu_FWD to amplify Mu or MuScr from pKD4_Mu or pKD4_MuScr for construction of Mu_origin 2 or MuScr_origin 2
yddW_Mu_FWD	ATATTCATACATTTTTATTAGGGATTTATGGCTGTTTAACGTGTAGGCTGGAGCTGCTTCG	Used in conjunction with yddW_Mu_REV to amplify Mu or MuScr from pKD4_Mu or pKD4_MuScr for construction of Mu_terminus 1 or MuScr_terminus 1

yddW_Mu_REV	CTAATAATCATGCTTACTTAAGTCAAATTAACCACACTTAGGGCGGTACACCCGATATG	Used in conjunction with yddW_Mu_FWD to amplify Mu or MuScr from pKD4_Mu or pKD4_MuScr for construction of Mu_terminus 1 or MuScr_terminus1
pfkB_Mu_FWD	GTATTCTTATTTCATTTTTTGAATAAGCATGTGGCGAAAACAGTGTAGGCTGGAGCTGCTTCG	Used in conjunction with pfkB_Mu_REV to amplify Mu or MuScr from pKD4_Mu or pKD4_MuScr for construction of Mu_terminus 2 or MuScr_terminus 2
pfkB_Mu_REV	GAGCTTTATTTAAAATTTTGCAGATAAATATATATAAATAAAAATCGGGCGGTACACCCGATATG	Used in conjunction with pfkB_Mu_FWD to amplify Mu or MuScr from pKD4_Mu or pKD4_MuScr for construction of Mu_terminus 2 or MuScr_terminus 2
**Primers used to verify gene perturbations**		
**Primer name**	**Sequence**	**Description**
pstS-int-fwd	AGGCTTGCTTCTGCAAACAC	Used in conjunction with glmS-int-rev to confirm chromosomal location of Mu or MuScr in Mu_origin 1and MuScr_origin 1, respectively
glmS-int-rev	GGTGATTGCACCGATCTTCT	Used in conjunction with pstS-int-fwd to confirm chromosomal location of Mu or MuScr in Mu_origin 1 and MuScr_origin 1, respectively
cat_R	GCAACTGACTGAAATGCCTC	Used in conjunction with Mu_int_rev to confirm chromosomal integration of Mu or MuScr
Mu_int_rev	CGCGCCACGGGATTTTTAAACC	Used in conjunction with cat_R to confirm chromosomal integration of Mu or MuScr

rsmG_ext_fwd	CAAACAATAAGTAGCCAAAAG	Used in conjunction with rsmG_int_rev to confirm chromosomal location of Mu or MuScr in Mu_origin 2 and MuScr_origin 2, respectively
rsmG_int_rev	AGCCCATTTCACTCTGTTGG	Used in conjunction with rsmG_ext_fwd to confirm chromosomal location of Mu or MuScr in Mu_origin 2 and MuScr_origin 2, respectively
yddW_ext_fwd	ATACTGAAAAGAAATAAGCG	Used in conjunction with yddW_int_rev to confirm chromosomal location of Mu or MuScr in Mu_terminus 1and MuScr_terminus 1, respectively
yddW_int_rev	TAAAAGCACGCCTCCAGAGT	Used in conjunction with yddW_ext_fwd to confirm chromosomal location of Mu or MuScr in Mu_terminus 1and MuScr_terminus 1, respectively
pfkB_ext_fwd	TGGTGTCAGCCGTAAGTGAG	Used in conjunction with pfkB_int_rev to confirm chromosomal location of Mu or MuScr in Mu_terminus 2 and MuScr terminus 2, respectively
pfkB_int_rev	ACCAGCGCACTGAGTTCTTT	Used in conjunction with pfkB_ext_fwd to confirm chromosomal location of Mu or MuScr in Mu_terminus 2 and MuScr_terminus 2, respectively
recD_int_fwd	TGCGCTTCTGTTGCATAAAC	Used in conjunction with recD_int_rev to confirm deletion of *recD*
recD_int_rev	TTTACAGAGCGGCGAAGATT	Used in conjunction with recD_int_fwd to confirm deletion of *recD*
argA_int_fwd	TCAAGGGGTGAAGTTCTGCT	Used in conjunction with recB_int_rev to confirm deletion of *recD* and the excision of *kan* cassette

recB_int_rev	TGAGATGTTTGCCGGTATGA	Used in conjunction with argA_int_fwd to confirm deletion of *recD* and the excision of *kan* cassette
kan_int_rev	ATGATGGATACTTTCTCGGCAGGAG	Used to confirm insertion of *FLAG-kan* or *kan* to the C-terminus of *gyrA* by Sanger sequencing
gyrA_ext_seq_rev	CCAAACTTTACCGTGCCCTA	Used in conjunction with gyrA_int_fwd4 to confirm insertion of *FLAG-kan* or *kan* to the C-terminus of *gyrA*
gyrA_int_fwd4	GGCGATAAAGTCGTCTCTCTGA	Used in conjunction with gyrA_ext_seq_rev to confirm insertion of *FLAG-kan* or *kan* to the C-terminus of *gyrA*
**Primers for ChIP-qPCR analysis**		
**Site**	**Forward primer (5′→3′)**	**Reverse Primer (5′→3′)**
Mu	CGACGTTGTAAAACGACGGCC	CGCGCCACGGGATTTTTAAACC
trkH	CTTTACCAGTATGAACCCGGTGG	CCAGAAAGTCGGGGTAAAGAGC
mobA	CTCACCAGCTTCCTGCTGC	ATTGAAGATTCACTGGCGGATTACC
nuoN	AGAGATCAGCACAACGATAC	CTACCTGCGCGTGGCGGTGA

Supplementary Table S3. Macrodomain boundary

**Macrodomain**(5, 6)	**Start***	**End***
NS-right	46418	603415
Right	603416	1206830

Ter	1206831	2181576
Left	2181577	2877824
NS-left	2877825	3759738
Ori	3759739	46417

* MD coordinates were adjusted to Mu-origin 1 genome

References

1. Kohanski MA, Dwyer DJ, Hayete B, Lawrence CA, Collins JJ. 2007. A Common Mechanism of Cellular Death Induced by Bactericidal Antibiotics. Cell 130:797–810.

2. Datsenko KA, Wanner BL. 2000. One-step inactivation of chromosomal genes in Escherichia coli K-12 using PCR products. Proc National Acad Sci 97:6640–6645.

3. Baba T, Ara T, Hasegawa M, Takai Y, Okumura Y, Baba M, Datsenko KA, Tomita M, Wanner BL, Mori H. 2006. Construction of Escherichia coli K-12 in-frame, single-gene knockout mutants: the Keio collection. Mol Syst Biol 2:2006.0008-2006.0008.

4. Sutormin D, Rubanova N, Logacheva M, Ghilarov D, Severinov K. 2018. Single-nucleotide-resolution mapping of DNA gyrase cleavage sites across the Escherichia coli genome. Nucleic Acids Res 47:1373–1388.

5. Valens M, Penaud S, Rossignol M, Cornet F, Boccard F. 2004. Macrodomain organization of the Escherichia coli chromosome. Embo J 23:4330–4341.

6. Lioy VS, Cournac A, Marbouty M, Duigou S, Mozziconacci J, Espéli O, Boccard F, Koszul R. 2018. Multiscale Structuring of the E. coli Chromosome by Nucleoid-Associated and Condensin Proteins. Cell 172:771-783.e18.
